# Combining P and Zn fertilization to enhance yield and grain quality in maize grown on Mediterranean soils

**DOI:** 10.1038/s41598-021-86766-2

**Published:** 2021-04-01

**Authors:** Antonio Rafael Sánchez-Rodríguez, María-Dolores Rey, Hasna Nechate-Drif, María Ángeles Castillejo, Jesús V. Jorrín-Novo, José Torrent, María Carmen del Campillo, Daniel Sacristán

**Affiliations:** 1 Department of Agronomy, University of Córdoba, Campus de Rabanales, Building, 14071 Córdoba, Spain; 2 Department of Agroforestry and Plant Biochemistry, Proteomics and Systems Biology, Biochemistry and Molecular Biology, University of Córdoba, Campus de Rabanales, Building C6, 14071 Córdoba, Spain

**Keywords:** Element cycles, Proteomics, Abiotic, Element cycles

## Abstract

The main aim of this study was to elucidate the effect of individual and joint fertilization with P and Zn on maize plants grown on typical Mediterranean soils with a limited Zn availability. For this purpose, we examined the effects of P and Zn fertilization individually and in combination on growth, yield and grain protein content in maize grown in pots filled with three different Mediterranean soils (LCV, FER and INM). Phosphorus and Zn translocation to grain was impaired, and aboveground dry matter and yield at harvest reduced by 8–85% (LCV and FER), in plants treated with Zn or P alone relative to unfertilized (control) plants. In contrast, joint fertilization with P and Zn enhanced translocation of these nutrients to grain and significantly increased aboveground dry matter (30% in LCV, 50% in FER and 250% in INM) and grain Zn availability in comparison with control plants. Also, joint application of both nutrients significantly increased grain P (LCV) and Zn (LCV and FER) use efficiency relative P and Zn, respectively, alone. Yield was increased between 31% in LCV and 121% in FER relative to control plants, albeit not significantly. Fertilization with P or Zn significantly influenced the abundance of specific proteins affecting grain quality (viz., storage, lys-rich and cell wall proteins), which were more abundant in mature grains from plants fertilized with Zn alone and, to a lesser extent, P + Zn. Sustainable strategies in agriculture should consider P–Zn interactions in maize grown on soils with a limited availability of Zn, where Zn fertilization is crucial to ensure grain quality.

## Introduction

Cereals (particularly rice, wheat, and maize) are used to feed human populations worldwide. In fact, cereals account for 55–70% of the total calories consumed in some Asian, African, Latin American and Caribbean countries^1^. Maize yields are strongly dependent on phosphorus (P) availability in soil. Although fertilizing soil with P increases its phytoavailability, only a small fraction (< 20%) of all P added remains available for plants in the short-term owing to the complexity of the P biogeochemical cycle. Also, a substantial amount of P is lost via leaching, run-off and erosion, and increases eutrophication in water bodies. These shortcomings have raised the need for sustainable strategies to improve P use efficiency in agriculture^2^. In addition, overusing P fertilizers does not increase crop yields; rather, it can have an adverse effect on the uptake of other essential nutrients such as zinc (Zn) by plants^3^ and thus reduce crop yield and quality.

It has been estimated that one-half of all arable land used to grow cereals in the world is under Zn deficiency owing to the alkaline pH of the soil among other factors^4^. Also, many rural populations in underdeveloped and developing countries have a low intake of Zn because their diet consists largely of cereals grown on soils with a low Zn availability, which eventually leads to health problems^5^. Zn fertilizers can temporarily alleviate Zn deficiency in cereals and help grow biofortified cereals to avoid it in plants and humans^6^. How soil Zn restrictions or deficiency influence cereal yields and grain quality in Southern Europe remains largely unknown, however.

Using chemical—and organic—inputs in agriculture increases crop yields but has deleterious side effects on the environment including eutrophication of water bodies, production of toxic and greenhouse gases, and loss of soil functionality. Sustainable intensification strategies aim at avoiding these problems by using land, water and energy resources in a sensible manner to ensure adequate food supply and security with provision for climate change and biodiversity^7^. However, sustainable intensification is hindered by gaps in existing knowledge about the mechanisms governing nutrient uptake and interactions, metabolite synthesis and nutrient translocation to edible crop parts (grains). Mediterranean soils typically have a limited P (and Zn) availability, a problem that is commonly addressed by fertilization with P at high rates^8^. This fertilization practice can further reduce Zn availability if the soil has a basic pH (around 8.0) and contains minerals such as CaCO_3_^4^. In any case, the implications of P–Zn interactions in Mediterranean soils are still poorly understood.

In nutritional terms, maize grains consist mainly of starch (70%) and protein (10%), and their quality depends largely on their protein content and composition. Maize proteins are mainly storage proteins and, especially, prolamins (also known as “zeins”), albumins, globulins and glutamines^9^. The zein fraction accounts for around 60% of all storage proteins; also, it has a low nutritional value for humans and monogastrics owing to the absence of essential amino acids such as lysine (lys) and tryptophan (trp)^9^. Unsurprisingly, plant breeders and agronomists have strived to develop maize lines with increased lys and trp contents^10^. Mertz et al.^11^ reported the most important discovery in maize breeding programmes: a high lysine *opaque2* maize mutant. However, the mutant had some undesirable properties such as high susceptibility to pathogens, low yields and a softy endosperm. These shortcomings were circumvented by developing Quality Protein Maize (QPM) varieties with favourable agronomic characteristics and a modified vitreous endosperm that retains increased amounts of lys and trp proteins. Lysine and tryptophan contents can be raised by using conventional breeding and transgenic methods, and also by supplying specific nutrients^12,13^. Grain protein content and quality can be improved by ensuring an appropriate nutrient balance in soil and plants.

Most existing studies on cereal grain proteins have been conducted on wheat-based N and its interactions with other nutrients such as zinc or sulphur and on rice-based micronutrients such as zinc, selenium and iron^14–16^. To our knowledge, only a few have addressed changes in the maize grain proteome by effect of nutrient supply, and even fewer have examined the effects of P–Zn interaction on grain protein content and, specifically, grain quality. In response, this work was undertaken to elucidate the effect of individual and joint fertilization with P and Zn on maize plants grown on three Mediterranean soils with a limited Zn availability with a view to improving plant growth, yield and grain quality (viz., grain Zn concentration and availability, and mature grain proteome profile). We hypothesized that applying Zn to the soils would increase maize yield and grain quality—particularly if Zn was delivered in combination with P—and applying P or Zn only would disrupt the balance between these nutrients in soil and plants, thereby reducing plant growth and yield, and grain quality.

## Methods

### Soil sampling

Samples of 200 kg were collected from the topsoil (5–20 cm depth, Ap horizon) of arable land in three agricultural fields from southern Spain. The first soil was an Alfisol (Rhodoxeralf^17^) from the province of Málaga (37° 10′ 32″ N, 4° 41′ 31″ W) and was named after the nearest town: Los Carvajales (LCV). The second soil, a Vertisol (Haploxerert^17^), was from the province of Córdoba (37° 42′ 13″ N, 4° 42′ 43″ W) and named FER (after Fernán Núñez). The third was an Inceptisol (Calci/Haploxerept^17^) from the province of Ciudad Real (39° 04′ 00′′ N, 3° 04′ 00′′ W) and named INM. The samples were passed through a 1 cm sieve to remove stones and homogenized in the field before they were air-dried for 1 week for use in pot experiments. An amount of 0.5 kg of each soil sample was ground to pass a 2 mm sieve and used in duplicate laboratory analyses (see Table S1 for information about soil properties, analytical methods and differences between soil properties).

### Pot experiments, experimental design, and treatments

Maize (*Zea mays* L. cv. ES ZOOM YG, provided by SAT Córdoba, www.satcordoba.es) was grown in pots (10 L volume, 23.5 cm depth, 27 cm diameter at the top and 20 cm at the base). The amount of soil used to fill each pot (9.5 kg of LCV or INM, or 8.5 kg of FER) was placed on an individual tray and homogeneously sprayed with 1.7 L (FER) or 1.9 L (LCV and INM) of P, Zn or P + Zn solution. The soil treatments were as follows: *C* (control; no P or Zn applied; 0 mg P/Zn kg^−1^); *P* (spraying with KH_2_PO_4_ at a rate of 40 mg P kg^−1^); *Zn* (spraying with ZnSO_4_·7H_2_O at 3 mg Zn kg^−1^); and *PZn* (spraying with the previous two solutions at the same individual rates). Following treatment, the samples were dried at 30 °C for 48 h and used to fill pots according to a completely randomised experimental design with 4 treatments and 4 replicates per soil and treatment combination (48 pots in total).

Then, three maize seeds were sown in each pot and the pots were immersed in trays containing 3.8 L (FER) or 4.4 L (LCV, INM)—45% of the soil volume was assumed to consist of pores—of modified Hoagland solution without P and Zn [viz., 5 mM Ca (NO_3_)_2_·4H_2_O, 5 mM KNO_3_, 2 mM MgSO_4_, 0.1 μM KCl, 50 μM H_3_BO_3_, 4 μM MnSO_4_·H_2_O, 0.1 μM CuSO_4_·5H_2_O and 6 μM Na_2_MoO_4_]. Iron sulphate (FeSO_4_) was added as source of Fe to the trays filled with calcareous soils (viz., 0.5 g L^−1^ to FER pots and 1.0 g L^−1^ to INM pots) to prevent Fe deficiency. After 24 h, the trays were removed and the pots allowed draining for 48 h before each pot, where the soil should be near field capacity, was weighed. After weighing, the pots were watered with deionised water according to plants requirement and supplied with modified Hoagland solution (mean of 7 mL per week but it was variable, i.e., up to 15–30 mL per week were added during anthesis and 0 mL after that and during grain maturity) to keep them near field capacity and supply them with nutrients other than P and Zn. Subsequently, the pots were kept in a growth chamber for 2 months (photoperiod 16 h day^−1^; light intensity 350 µmol m^−2^ s^−1^; 25 °C during the day and 20 °C at night; relative humidity 55%) and then in a greenhouse under identical conditions for the remainder of the experiment. Ten days after sowing (DAS), two seedlings were cut and removed from each pot, the experimental unit being one maize plant per pot.

### Plant measurements and determinations

Plant height, stem perimeter 2 cm above the soil and leaf thickness of the completely expanded penultimate leaf as measured with a vernier caliper were obtained on a weekly basis until grain maturity (160 DAS). Leaf chlorophyll index in the last two completely expanded leaves (LCI) with the aid of an SPAD 502 Portable Chlorophyll Meter (Minolta Camera Co., Osaka, Japan) every week until grain filling (Table S2, Figs S1 and S2). Plants were cut off at grain maturity and dried at 65 °C for 72 h. Then, their different parts (leaf, stem, corncob, corncob leaf and grain) were separated and weighed, and grains counted; also, each part was grounded in a mill for digestion with nitric and perchloric acids. Phosphorus was determined with the Molybdenum Blue method and Zn by atomic absorption spectroscopy.

P and Zn uptake were calculated by multiplying the biomass of each plant part by its content in P and Zn, respectively. These individual values were divided by their combined value for P or Zn—separately and for each plant—to calculate the P and Zn distribution in plant, respectively. Finally, grain and plant P use efficiency (PUE, treatments *P* and *PZn*) and Zn use efficiency (ZnUE, treatments *Zn* and *PZn*) were calculated from the following equations:

1 $$PUE\; \left( \% \right) = 100 \times \frac{{Puptake \;\left( {P, Zn or PZn} \right) - Puptake\; \left( C \right)}}{Papplied}$$

2 $$ZnUE\; \left( \% \right) = 100 \times \frac{{Znuptake \;\left( {P, Zn or PZn} \right) - Znuptake\; \left( C \right)}}{Znapplied}$$

where *P*_uptake_ (*P*,* Zn or PZn*)*, P*_uptake_ (*C*), *Zn*_uptake_ (*P*,* Zn or PZn*) and *Zn*_uptake_ (*C*) are the P and Zn uptake at harvest (grain and whole plant) for the maize plants fertilized with P (*P*), Zn (*Zn*) and P + Zn (*PZn*), and the control plants (*C*); and *P*_applied_ and *Zn*_applied_ are the amounts of P and Zn, respectively, sprayed to the soil in each case. Finally, the mole ratio of P to Zn in each plant part was used as a proxy for Zn availability in each plant part (particularly in grain).

### Shotgun proteomic analysis of grains

Grain protein was extracted according to the trichloroacetic acid (TCA)/acetone–phenol protocol^18^ and its concentration determined with the Bradford method (BioRad, Hercules, CA, USA), using bovine serum albumin (BSA) as standard^19^. For protein sample cleaning, 60 µg of BSA protein equivalent from each biological replicate of each treatment was subjected to sodium dodecyl sulfate polyacrylamide gel electrophoresis (SDS-PAGE) and the only resulting band digested with trypsin (12.5 ng µL^−1^) (Sequencing grade, Promega, Madison, WI, USA)^19^. A shotgun (Liquid Chromatography with tandem mass spectrometry, LC–MS/MS) analysis was then performed according to Castillejo et al^20^.

All raw data were processed with the software Proteome Discoverer v 2.1.0.81 (Thermo Scientific, San Jose, CA, USA). MS2 spectra were searched with SEQUEST engine against the specie specific *Z. mays* from UniPrtoKB database. For identification, peptides were grouped into proteins according to the law of parsimony and filtered to FDR = 0.01 and *X*_Corr_ ≥ 2. The following parameters were allowed: a maximum of 2 miss cleavages of trypsin, carbamidomethylation of cysteines as a fixed modification and oxidation of methionine as a variable modification. Proteins were quantified from peak areas, which were normalized by the combined total area for each sample. The software Proteome Discoverer creates protein groups from identified peptide spectrum matches (PSMs) and considers the presence of proteins with shared peptides. The software excludes all protein groups having no unique peptides (i.e., peptides that are not shared with any other protein). Shared peptides are quantified by dividing their assigned values by the number of proteins in which they are present. Those proteins with at least 2 peptides score higher than 2 and a sequence coverage higher than 15% is considered for further analysis. The criteria used to consider protein a change were as follows: (a) protein consistently present or absent in all three replicates for a condition; (b) the change was at least twofold; and (c) there were statistically significant differences (One-way ANOVA, *p* < 0.05) between treatments. A Venn diagram was constructed according to Oliveros^21^. Also, a Gene Ontology (GO) analysis was used to retrieve differentially abundant proteins (DAPs) from the UniProt-GOA database on 10 April 2020 for functional annotation in terms of biological process, molecular function and cellular component. The molecular functions for the DAPs were determined by using MERCATOR (http://www.plabipd.de/portal/‌mercator-sequence-annotation/).

The mass spectrometry proteomics data have been deposited to the ProteomeXchange Consortium via the PRIDE partner repository with the dataset identifier PXD021503^22^.

### Statistical analysis

Repeated measures analysis of variance (RM ANOVA; time and treatments *C*, *P*, *Zn* and *PZn*) was performed for the variables that were weekly measured (viz., plant height, stem perimeter, leaf thickness and number of leaves); whereas one-way ANOVA with four treatments (*C*, *P*, *Zn* and *PZn*) was used with the variables measured or calculated at harvest and those that were measured weekly when the interaction time × treatment was significant. The RM and one-way ANOVAs were applied independently to each soil. When differences were significant (*p* < 0.05), the Fisher’s Least Significant Differences (LSD) post-hoc test was used to separate means. A completely randomised design was used in all cases. The previous analyses were performed with the software Statistix v. 10.0 (Analytical Software, Tallahassee, FL, USA). A multivariate analysis (principal component analysis, PCA) based on a data correlation matrix with principal components (PCs), a heatmap and a one-way ANOVA (*p* < 0.05) were also performed, using the software R′^23^, https://jcoliver.github.io/learn-r/006-heatmaps.html and the pRocessomics R package (https://github.com/Valledor/pRocessomics), respectively, in the proteomic analysis.

## Results

### Plant growth

Maize plants grown on soil INM had the smallest plant height and stem perimeter—by exception, *PZn* plants reached similar or even greater heights than those grown on LCV and FER. Overall, treatment *PZn* had a positive effect on plant height and stem perimeter, which exhibited the greatest values among treatments (Fig. S1 and Table S2). Treatment *P* had an adverse impact on plant height but increased stem perimeter relative to *C* in most measurements; by contrast, *Zn* had a negligible effect (Fig. S1). Leaf chlorophyll index (LCI) was similar among plants grown on the three soils, the differences between treatments only being significant in FER and INM. Although LCI was significantly increased by treatments *P*, *Zn*, and *PZn* in relation to *C* at least once, the increase was more consistent with *PZn* than with the other treatments (Table S2 and Fig. S2). Aboveground dry matter (straw and grain) and yield (grain weight) at harvest were greater in LCV and FER than they were in INM (Table 1). *PZn* increased aboveground dry matter in the three soils relative to the other treatments (*C*, *P* and *Zn*) and also grain yield relative to the *P* and *Zn* treatments. However, only two maize plants grown on INM produced any grain, both under *PZn*. Although the number of seeds per plant and thousand grain weight (TGW) were greatest in *PZn* treated plants, there were significant differences in the number of seeds the plants grown on LCV only (*p* = 0.022; Table 1).

**Table 1:** Aboveground dry matter (straw and grain), yield (grain), number of seeds per plant and thousand grain weight (TGW) at harvest of maize plants (mean ± standard error, *n* = 4 except for grain, yield gain, number of seeds and TGW in soil INM, where it was *n* = 2 in treatment *PZn*) as a function of treatment for each soil (LCV, FER and INM).

Treatment	Aboveground biomass (g)	Grain (g)	Seed number (No)	TGW (g)
**LCV**				
*C*	124.5 ± 11.1 b	44.6 ± 15.1 ab	146.3 ± 50.0 ab	230 ± 68
*P*	101.5 ± 5.3 b	7.0 ± 6.0 b	21.8 ± 17.7 b	144 ± 74
*Zn*	111.4 ± 2.7 b	6.7 ± 2.0 b	29.5 ± 11.3 b	242 ± 35
*PZn*	162.0 ± 17.1 a	58.4 ± 18.8 a	224.8 ± 72.5 a	258 ± 3
*p* value	**0.008**	**0.025**	**0.022**	0.573
**FER**				
*C*	120.2 ± 9.0 b	28.6 ± 13.9 ab	90.8 ± 46.0	320 ± 12
*P*	111.0 ± 6.0 b	10.5 ± 7.0 b	32.3 ± 22.1	307 ± 3
*Zn*	121.3 ± 11.8 b	26.4 ± 16.5 ab	96.5 ± 60.7	290 ± 16
*PZn*	179.8 ± 7.9 a	63.1 ± 14.8 a	223.5 ± 61.6	292 ± 13
*p* value	**< 0.001**	**0.010**	0.104	0.412
**INM**				
*C*	31.0 ± 3.9 b	0.0 ± 0.0	0.0 ± 0.0	na
*P*	39.8 ± 1.4 b	0.0 ± 0.0	0.0 ± 0.0	na
*Zn*	27.6 ± 4.1 b	0.0 ± 0.0	0.0 ± 0.0	na
*PZn*	107.6 ± 6.6 a	2.3 ± 2.0	6.3 ± 5.0	165 ± 86
*p* value	**< 0.001**	na	na	na

Significant *p* values are in bold to indicate significant differences.

*p* is the probability level of the one-way ANOVA. Different letters indicate significant differences between treatments according to the posthoc LSD test.

*C*: no P or Zn was added; *P*: fertilization with 40 mg P kg^−1^ but no Zn; *Zn*: fertilization with 3 mg Zn kg^−1^ but no P; *PZn*: fertilization with 40 mg P kg^−1^ and 3 mg Zn kg^−1^. na: non-available.

### Phosphorus and zinc accumulation, and nutrient use efficiency

As can be seen from Table 2, supplying the soil with P (*P* and *PZn* plants) or Zn (*Zn* and *PZn* plants) increased plant P and Zn uptake, respectively,—note the marked increase in the plants grown on INM. The greatest P and Zn gains in grains relative to treatment *C* were provided by *PZn* (36% for P in LCV and 40% in FER, and 91% for Zn in LCV and 157% in FER, Table 2). The application of only one of the two nutrients to LCV and FER, produced the lowest grain P and Zn uptake (except for *Zn* plants grown in FER). Finally, the previous differences were especially reflected in plant ZnUE (all three soils), and grain PUE and ZnUE (LCV and FER). Applying both nutrients (*PZn* plants) resulted in greater values than supplying either alone (*P* and *Zn* plants; Table 2).

**Table 2:** Phosphorus and zinc uptake (plant and grain), and phosphorus and zinc use efficiency (plant and grain PUE and ZnUE, respectively) at harvest (mean ± standard error, *n* = 4) as a function of the treatment for each soil (LCV, FER and INM).

Treatment	Plant P uptake (mg)	Grain P uptake (mg)	PUE_plant_ (%)	PUE_grain_ (%)	Plant Zn uptake (µg)	Grain Zn uptake (µg)	ZnUE_plant_ (%)	ZnUE_grain_ (%)
**LCV**								
*C*	250.6 ± 17.7 bc	153.7 ± 52.6 ab	na	na	2107 ± 187 b	1043 ± 359 ab		
*P*	276.3 ± 1.3 ab	32.6 ± 28.3 b	6.8 ± 0.3	− 31.9 ± 7.5 b	1558 ± 135 b	266 ± 224 b	na	na
*Zn*	197.1 ± 20.1 c	24.4 ± 10.8 b	na	na	4093 ± 316 a	286 ± 116 b	7.0 ± 1.1	− 2.7 ± 0.4 b
*PZn*	322.8 ± 36.1 a	209.4 ± 63.2 a	19.0 ± 9.5	14.6 ± 16.6 a	4793 ± 365 a	1996 ± 613 a	9.4 ± 1.3	3.3 ± 2.2 a
*p* value	**0.014**	**0.027**	0.247	**0.044**	**< 0.001**	**0.022**	0.197	**0.034**
**FER**								
*C*	132.5 ± 22.4 b	74.6 ± 30.2	na	na	2212 ± 261 c	830 ± 383 b		
*P*	239.4 ± 32.1 a	39.2 ± 26.5	31.4 ± 9.4	− 10.4 ± 7.8	2010 ± 150 c	361 ± 243 b	na	na
*Zn*	159.5 ± 17.5 b	76.2 ± 36.0	na	na	4448 ± 217 b	626 ± 341 b	9.6 ± 0.8 b	1.0 ± 1.3 b
*PZn*	178.0 ± 22.0 ab	104.2 ± 25.0	13.4 ± 6.5	8.7 ± 7.4	5520 ± 65 a	2131 ± 377 a	13.8 ± 0.3 a	6.9 ± 1.5 a
*p *value	**0.048**	0.516	0.165	0.125	**< 0.001**	**0.014**	**0.003**	**0.025**
**INM**								
*C*	29.9 ± 7.5 b	0	na	na	612 ± 146 c	0	na	
*P*	107 ± 6.6 a	0	20.0 ± 1.9	0	627 ± 59 c	0	na	na
*Zn*	25.3 ± 7.7 b	0	na	na	1368 ± 289 b	0	2.6 ± 1.0 b	na
*PZn*	86.0 ± 8.3 a	17.0 ± 15.1	14.8 ± 2.2	4.5 ± 4.0	3917 ± 246 a	125 ± 111	11.5 ± 0.9 a	0.4 ± 0.4
*p * value	**< 0.001**	na	0.094	0.303	**< 0.001**	na	**< 0.001**	0.304

Significant *p * values are in bold to indicate significant differences.

*p* value: probability level of the one-way ANOVA. Different letters indicate significant differences between the treatments, according to the posthoc LSD test.

*C*: control treatment (no P or Zn were added); *P*: phosphorus fertilization (40 mg P kg^−1^; no Zn was added); *Zn*: zinc fertilization (3 mg Zn kg^−1^; no P was added); *PZn*: phosphorus plus zinc fertilization (40 mg P kg^−1^ and 3 mg Zn kg^−1^). na: non-available.

### Phosphorus and zinc distribution within plants

**Figure 1:**
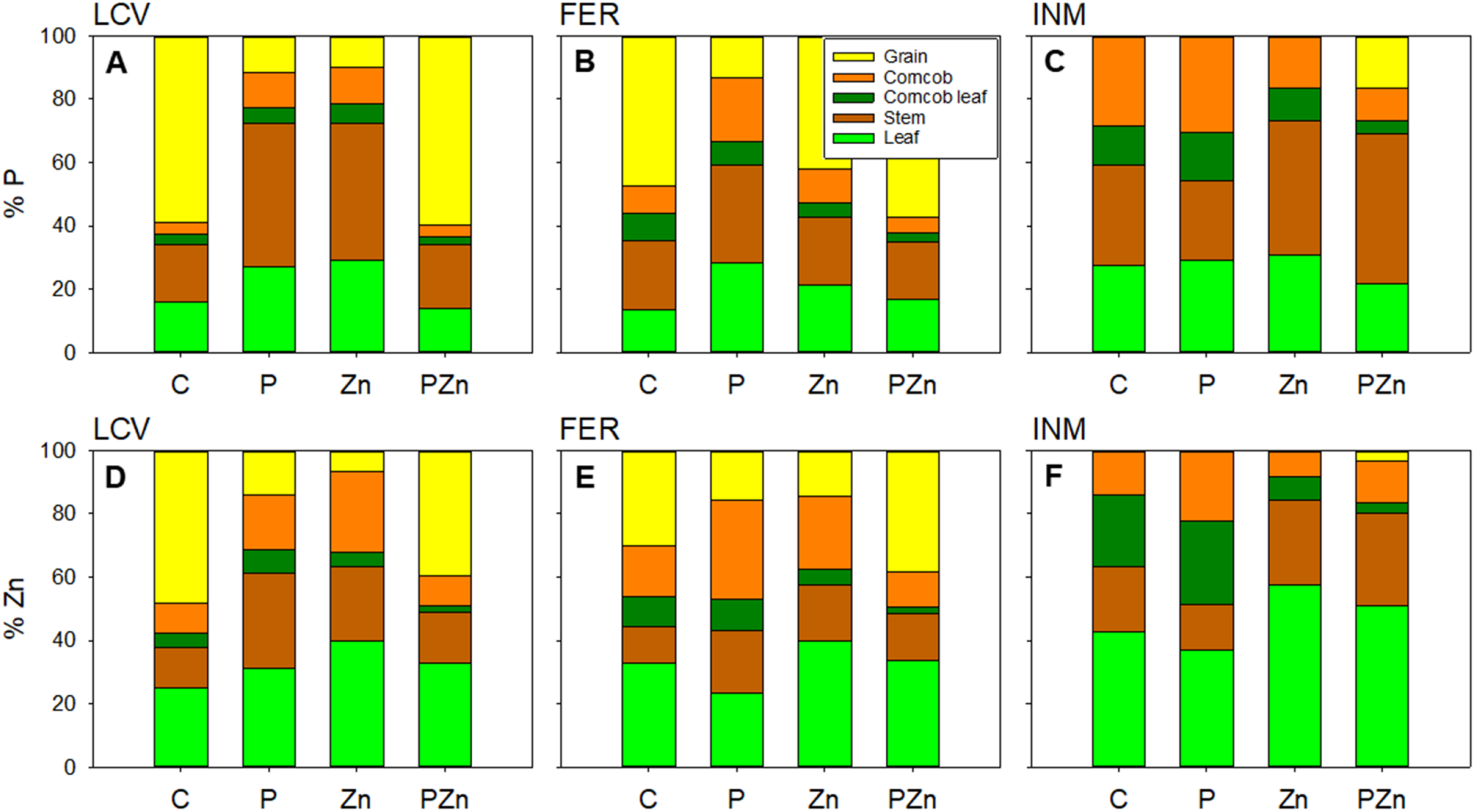
P and Zn distribution (%) in different plant parts (mean, *n* = 4) at harvest by treatment (*C*: no P or Zn was added; *P*: fertilization with 40 mg P kg^−1^ but no Zn; *Zn*: fertilization with 3 mg Zn kg^−1^ but no P; *PZn*: fertilization with 40 mg P kg^−1^ and 3 mg Zn kg^−1^). (**A**, **D**), soil LCV. (**B**, **E**), soil FER. (**C**, **F**), soil INM.

A similar distribution of P and Zn in plant parts was observed in LCV and FER but not in INM (Fig. 1). Also, treatments *C* and *PZn* led to similar P and Zn distribution patterns in the different parts of plants grown on LCV and FER, where a substantial proportion of P accumulated in grains with treatment *C* (59 and 47%, respectively) and *PZn* plants (60 and 58%, respectively) relative to *P* and *Zn* plants (< 14% in all except P in *Zn* plants grown on FER, which accumulated around 42%). The Zn pattern was similar, with *C* plants (49 and 30%, respectively, in LCV and FER) and *PZn* plants (40 and 39%, respectively, in LCV and FER) accumulating the highest proportions of Zn in grain, and *P* and *Zn* plants the lowest (< 16%). In *P* and *Zn* plants, these two nutrients accumulated more markedly in stem or leaf, followed by corncob, than they did with the other treatments (Fig. 1). The grains of maize plants grown on INM accumulated no P or Zn except in those that were fertilized jointly with both nutrients (i.e., *PZn* plants, with 16.4% of P accumulation and 3.2% of Zn accumulation). In plants grown on INM, P accumulated similarly in leaf, stem and corncob with treatments *PZn*, *C* and *P* (around 30% in each plant part); by contrast, P tended to accumulate in stem with treatments *Zn* (43%) and *PZn* (47%). Supplying the soils with Zn (*Zn* and *PZn* plants) resulted in increased accumulation of this element in leaf and stem, but also in decreased accumulation in corncob and corncob leaf relative to *C* and *P* plants (Fig. 1).

In line with these results, the P:Zn mole ratio in leaf, stem, corncob, and corncob leaf was generally increased by *P*, and reduced by both *Zn* and *PZn*, relative to *C* (Table 3).

**Table 3:** Phosphorus to zinc mole ratio for each plant part of the maize crop at harvest (mean ± standard error, *n* = 4 except for grain in INM, where *n* = 2) as a function of treatment for each soil.

Treatment	Leaf	Stem	Corncob leaf	Corncob	Grain
**LCV**					
*C*	149 ± 36 b	347 ± 23 b	164 ± 13 b	57 ± 11 b	311 ± 4 a
*P*	353 ± 27 a	615 ± 19 a	281 ± 17 a	189 ± 55 a	237 ± 27 b
*Zn*	80 ± 11 bc	189 ± 17 c	147 ± 13 b	32 ± 6 b	170 ± 17 c
*PZn*	55 ± 19 c	168 ± 27 c	139 ± 8 b	29 ± 6 b	223 ± 4 b
*p* value	**< 0.001**	**< 0.001**	**< 0.001**	**0.004**	**< 0.001**
**FER**					
*C*	69 ± 29 b	189 ± 42 b	132 ± 19 b	29 ± 13 b	202 ± 21 a
*P*	326 ± 44 a	521 ± 189 a	208 ± 11 a	95 ± 19 a	225 ± 2 a
*Zn*	44 ± 17 b	76 ± 17 b	69 ± 6 c	17 ± 8 b	273 ± 50 a
*PZn*	34 ± 4 b	74 ± 19 b	113 ± 11 b	21 ± 2 b	103 ± 19 b
*p* value	**< 0.001**	**0.021**	**< 0.001**	**0.002**	**0.010**
**INM**					
*C*	97 ± 29 b	221 ± 48 b	74 ± 6 b	84 ± 21	na
*P*	372 ± 69 a	767 ± 57 a	258 ± 46 a	116 ± 13	na
*Zn*	23 ± 4 b	71 ± 13 c	67 ± 6 b	59 ± 21	na
*PZn*	18 ± 2 b	75 ± 8 c	86 ± 32 b	46 ± 19	288 ± 2
*p* value	** < 0.001**	** < 0.001**	** < 0.001**	0.090	na

Significant *p* values are in bold to indicate significant differences.

*p* is the probability level of the one-way ANOVA. Different letters indicate significant differences among treatments according to the post-hoc LSD test.

*C*: no P or Zn was added; *P*: fertilization with 40 mg P kg^−1^ but no Zn; *Zn*: fertilization with 3 mg Zn kg^−1^ but no P; *PZn*: fertilization with 40 mg P kg^−1^ and 3 mg Zn kg^−1^. na: non-available.

### Phosphorus and zinc grain concentrations and P:Zn mole ratio

**Figure 2:**
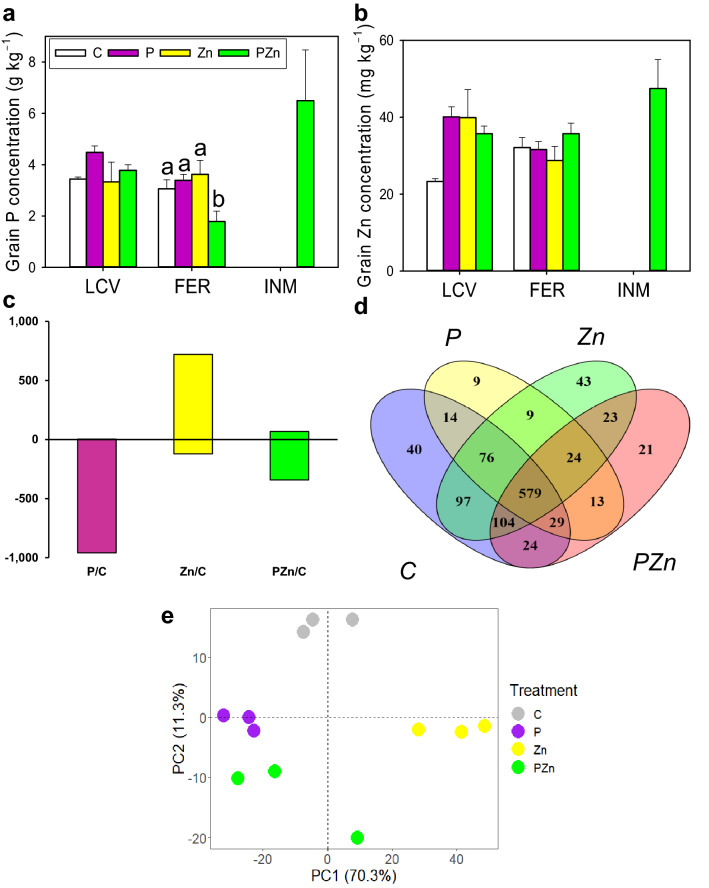
Grain P (**A**) and Zn (**B**) concentration (mean ± standard error, *n* = 4) at harvest as a function of soil and treatment. (*C*: no P or Zn was added; *P*: fertilization with 40 mg P kg^−1^ but no Zn; *Zn*: fertilization with 3 mg Zn kg^−1^ but no P; *PZn*: fertilization with 40 mg P kg^−1^ and 3 mg Zn kg^−1^). Different letters indicate significant differences between treatments according to the post-hoc LSD test. No letter means there were no significant differences. (**C**) Ratio of grain proteins whose abundance at harvest was greater or less than with treatment *C*. Number of up- and down-accumulated proteins represented as positive and negative values, respectively. (**D**) Venn diagrams showing all differentially abundant grain proteins altered by fertilization with P and/or Zn. (**E**) PCA exposing differences in grain proteomic profile between treatments. The proteomic analysis was conducted on three biological replicates (*n* = 3).

Grain P concentrations at harvest were similar in maize plants grown on LCV and FER, but slightly higher in those grown on INM—only *PZn* plants produced any grains in this soil. The P concentrations in grain from plants grown on FER were significantly lower (*p* = 0.030) with *PZn* (1.79 g kg^−1^) than they were with *C* (3.06 g kg^−1^),* P* (3.39 g kg^−1^) and *Zn* (3.62 g kg^−1^) plants. By contrast, such concentrations were essentially similar for plants grown on LCV (Fig. 2A). Grain Zn concentrations were lower in *C* plants grown on LCV than in those grown on FER; also, though not significantly, such concentrations were 58.5% (LCV) and 11% higher (FER) with treatments *P* and *Zn* than they were with *C* (Fig. 2B). In addition, the P:Zn mole ratio in grain was significantly lower in *Zn* plants, followed by *P* and *PZn* plants, than it was in *C* plants grown on LCV (*p* < 0.001), but only in *PZn* plants among those grown on FER (*p* = 0.010; Table 3).

### Protein content and profile in maize grains

Mature grains of plants grown on soil LCV were selected for proteomic analysis based on the grain Zn concentration in *C* plants, which was low relative to soil FER and to the other treatments (*P*, *Zn* and *PZn*). A total of 1756 proteins out of 3427 were identified (Table S3). No significant differences (*p* = 0.144) in total protein content between fertilizer treatments were observed. Such a content was (411 ± 145), (780 ± 66), (443 ± 252) and (509 ± 229) µg with treatments *C*, *P*, *Zn* and *PZn*, respectively. Only the most abundant proteins identified in all treatments (Table 4, Table S4) were used for further study.

**Table 4:** List of differentially accumulated proteins altered by the presence of P and Zn in mature grains as grouped by most abundant proteins, seed storage proteins and trp- and lys-rich proteins. DAPs were determined according to statistically significant changes between samples by ANOVA (*p* < 0.05) and fold ≥ 2 or ≤ 0.5. The ID, description, number of peptides (≥ 2), score (≥ 2) and coverage (≥ 15%), relative abundance (%), *p* (< 0.05) and ratio (fold ≥ 2 or ≤ 0.5) of each protein are shown. The relative abundance (%) was calculated from the combined abundance of all treatments for each protein divided by the combination of proteins included in each treatment. The most abundant proteins, seed storage proteins and trp- and lys-rich proteins are listed in Table S4.

Protein ID	Description	No. of peptides	Score Sequest	Coverage	%	*p *value	Ratio		
							P/C	Zn/C	PZn/C
**Most abudant proteins**									
Q7M1Z8	Globulin-2	34	9504.25	74.22	15.39	*0.0243*	0.30	1.94	0.89
K7W272	Vicilin-like seed storage	31	5143.54	59.05	3.50	*0.0351*	0.50	2.68	1.27
A0A1D6K1B9	Hydroxyproline-rich glycoprotein family protein	25	3469.82	73.83	2.48	*0.0327*	0.50	2.88	1.26
A0A1D6F0W7	NAD(P)-binding Rossmann-fold superfamily protein	25	2708.60	74.86	2.47	*0.0304*	0.49	2.59	1.13
P15590	Globulin-1 S allele	44	8140.84	64.75	1.80	*0.0022*	0.26	3.31	1.34
A0A1D6LER3	Glyceraldehyde-3-phosphate dehydrogenase	23	2381.58	79.94	1.02	*0.0363*	0.45	2.98	1.27
B4FFZ9	Oil body-associated protein 1A	12	882.02	63.87	0.83	*0.0395*	0.50	2.36	1.08
K7V794	Enolase 1	31	2874.43	80.49	0.75	*0.0386*	0.51	3.10	1.46
K7VJF3	Heat shock 70 kDa protein 5	46	2773.17	72.24	0.69	*0.0023*	0.27	3.33	0.46
B6UH67	Late embryogenesis abundant protein D-34	13	1494.92	57.52	0.59	*0.0099*	0.43	3.11	0.95
B6SK87	rRNA N-glycosidase	18	1375.68	74.42	0.57	*0.0169*	0.33	3.10	1.18
A0A1D6NT56	Sucrose synthase	45	2475.12	54.50	0.51	*0.0352*	0.28	2.59	0.85
**Seed storage proteins**									
Q7M1Z8	Globulin-2	34	9504.25	74.22	15.39	*0.0243*	0.30	1.94	0.89
P15590	Globulin-1 S allele	44	8140.84	64.75	1.80	*0.0022*	0.26	3.31	1.34
A0A1R3QMY2	50kD gamma zein	7	552.98	36.04	0.43	*0.0292*	0.39	2.32	0.48
P04706	Glutelins	2	149.51	12.11	0.03	*0.0022*	1.39	0.36	0.93
P04701	Zein	2	75.44	26.22	0.03	–	1.44	0.00	3.11
A0A1D6KYZ7	Globulin-1 S allele	24	2581.57	81.00	0.02	–	0.00	3.31	0.00
**Trp- and Lys-rich proteins**									
K7W272	Vicilin-like seed storage protein	31	5143.54	59.05	3.50	*0.0373*	0.55	2.76	2.74
A0A1D6LER3	Glyceraldehyde-3-phosphate dehydrogenase (EC 1.2.1.-)	23	2381.58	79.94	1.02	*0.0002*	0.33	3.48	0.00
A0A1D6NT56	Sucrose synthase (EC 2.4.1.13)	45	2475.12	54.50	0.51	*0.0243*	0.36	1.23	0.94
Q946V2	Legumin 1 (Legumin1)	23	1511.43	63.98	0.45	*0.0000*	0.24	2.95	0.37
A0A1D6K268	Vicilin-like seed storage protein	16	1089.95	38.52	0.36	–	–	–	–
Q5EUE1	Protein disulfide-isomerase (EC 5.3.4.1)	37	2027.87	71.40	0.34	*0.0334*	0.28	2.21	0.51
B4FAL9	Fructose-bisphosphate aldolase (EC 4.1.2.13)	35	2480.04	85.63	0.24	*0.0143*	0.31	3.73	0.90
A0A1D6KL30	Sorbitol dehydrogenase	18	961.14	68.03	0.20	*0.0007*	0.25	1.72	1.24
K7UUB7	Elongation factor 1-alpha	21	2688.99	57.05	0.18	–	0.29	2.78	0.68
A0A1D6FW13	Actin-7	19	1169.94	54.85	0.11	–	0.00	1.61	3.24
O50018	Elongation factor 1-alpha	20	1972.30	53.24	0.10	*0.0270*	0.29	0.94	0.79
Q5EUE0	Protein disulfide-isomerase (EC 5.3.4.1)	27	766.07	54.88	0.10	*0.0365*	0.58	1.40	1.89
B6UHJ4	Elongation factor 1-alpha	20	1642.73	53.24	0.09	–	0.00	1.33	2.57
B6SMQ5	Triose phosphate isomerase5	13	412.68	63.67	0.08	*0.0031*	1.09	1.49	5.15
A0A1D6K2D7	Sucrose synthase (EC 2.4.1.13)	35	758.50	45.11	0.07	*0.0241*	0.64	3.67	1.90
A0A1D6LZ74	Protein disulfide isomerase7	18	426.66	50.53	0.02	*0.0350*	0.72	2.69	1.96
B4FVB1	Actin-7	16	780.68	56.50	0.02	*0.0263*	0.26	1.45	1.42
Q5EUD5	Protein disulfide isomerase8	11	241.78	36.45	0.02	*0.0351*	0.47	3.81	1.01
B4FS35	Indole-3-glycerol phosphate synthase chloroplastic	9	194.28	29.24	0.02	*0.0373*	0.39	2.85	1.15
B6SLV6	16.9 kDa class I heat shock protein 3	4	122.89	38.26	0.01	–	–	–	–
B6TWN7	Elongation factor 1-alpha	17	826.50	39.15	0.01	*0.0107*	0.53	2.35	1.90
B6TQ08	Actin-1	18	894.63	66.05	0.01	*0.0363*	1.84	1.52	6.64
B4FAK8	Calnexin homolog2	11	227.60	26.69	0.01	*0.0412*	0.49	2.20	1.34
B4FQ44	l-tryptophan–pyruvate aminotransferase 1	5	92.76	16.01	0.01	*0.0339*	0.19	3.33	0.80
B4FRH8	Actin-7	18	905.21	66.05	0.01	*0.0033*	0.45	1.99	2.09
A0A1D6QSB0	Glyceraldehyde-3-phosphate dehydrogenase (EC 1.2.1.-)	14	519.20	53.78	0.01	*0.0012*	0.28	0.83	0.76
A0A1D6L7S0	Tryptophan synthase alpha chain chloroplastic	4	33.61	23.72	0.00	–	0.00	2.61	1.00
C0P6F8	Sucrose synthase (EC 2.4.1.13)	15	264.09	20.47	0.00	*0.0359*	0.64	3.50	1.39
K7TR93	Tryptophan synthase	6	36.29	17.95	0.00	–	0.62	0.43	0.00
Q5EUD1	Protein disulfide isomerase12	7	12.03	16.56	0.00	*0.0352*	0.87	1.11	1.24
A0A1D6ERC4	Protein disulfide-isomerase like 2–2	10	204.96	32.35	0.00	*0.0025*	0.42	2.15	0.78
A0A1D6M1H2	Elongation factor 1-alpha	8	555.30	51.09	0.00	–	0.36	3.12	1.60

Italics are used to differentiate the *p *values.

Qualitative DAPs did not show *p *value data and qualitative DAPs without protein abundance in the control treatment did not show relative abundance data.

*C*: no P or Zn was added; *P*: fertilization with 40 mg P kg^−1^ but no Zn; *Zn*: fertilization with 3 mg Zn kg^−1^ but no P; *PZn*: fertilization with 40 mg P kg^−1^ and 3 mg Zn kg^−1^. na: non-available.

The most abundant protein was Globulin-2 (Q7M1Z8), with 15.39%, followed by vicilin-like seed storage protein (K7W272), with 3.50%, and 16.9 kDa class I heat shock protein 1 (B6SIX0), with 3.27%. The other most abundant proteins ranged from 0.5 to 2.5% in abundance (Table 4, Table S4). Among them, 96% up-accumulated with treatment *Zn*, 58% down-accumulated with *P* and 17% up-accumulated with *PZn* (Table 4, Table S4). Also, 60% exhibited significant differences (*p* < 0.05) between treatments and all were more abundant with *Zn*—by exception, Enolase 1 was more abundant with *Zn* and *PZn*.

A total of 1106 DAPs out of 1758 differed significantly between treatments; differences were qualitative (presence or absence in at least one treatment) in 527 of them and quantitative (*p* < 0.05) in 579 (Table S3). The greatest number of up-accumulated DAPs (721) was observed with *Zn* and that of down-accumulated DAPs (959) with *P* (Fig. 2C). Of the DAPs exhibiting qualitative differences, 40, 9, 43 and 21 were only found in *C*, *P*, *Zn* and *PZn* plants, respectively (Fig. 2D). The PCA on the grain proteomic profile separated each treatment and allowed all biological replicates to be grouped (Fig. 2E). PC1 explained 70.3% of the variance and suggested differences between treatment *Zn* and all others. On the other hand, PC2 explained 11.3% of the variance and suggested differences between *C* and the treatments including nutrients. Based on the Uniprot-GO annotations (Table S5), most DAPs involved in biological processes were translation, folding or refolding proteins. By contrast, most DAPs performing molecular functions were ATP binding, RNA binding or ribosomal structural constituent proteins. On the other hand, the proteins belonging to cellular components were mainly associated to the cytoplasm, cytosol or nucleus. Molecular function analysis revealed that the major pathways were protein synthesis (28%), RNA synthesis (5.54%), amino acid metabolism (5.54%) and stress response (5.29%) (Fig. S3).

A total of 13 proteins (6 zeins, 5 globulins, 1 albumin and 1 glutelin) were maize grain storage proteins (Table 4, Table S4). Two globulins (Q7M1Z8 and P15590), and one zein (A0A1R3QMY2,) differed significantly (*p* < 0.05) between treatments (Table 4). Also, one globulin (A0A1D6KYZ7) and one zein (P04701) were qualitative DAPs (Table 4). Relative to *C* plants, Q7M1Z8 was down-accumulated in *P*, P15590 up-accumulated in *Zn* but down-accumulated in *P*, A0A1R3QMY2 up-accumulated in *PZn*, P04701 up-accumulated in *PZn* but down-accumulated in *Zn*, and A0A1D6KYZ7 was only present in *Zn* and down-accumulated in *P* and *PZn* (Table 4).

Two DAPs were trp-rich proteins and 24 lys-rich proteins (Table 4). Three of the trp-rich proteins were up-accumulated in *Zn* but down-accumulated in *P*; also, one was down-accumulated in both *Zn* and *PZn* (Table 4, Table S4). In the lys-rich protein group, 53% were up-accumulated in *Zn*, 60% down-accumulated in *P*, 23% up-accumulated in *PZn* and 10% down-accumulated in the same treatment (Table 4).

## Discussion

The differences in growth and yield in maize grown on the three soils can be ascribed to differences in soil properties. Thus, although the three soils have a basic soil pH (ca. 8 due to their content in CaCO_3_), INM and FER contained greater amounts of CaCO_3_ than did LCV. The plants grown on INM found it more difficult to grow owing to the sandy texture of this soil, its low fertility—it had the lowest OM, available Zn and available Fe contents, and CEC—and its very high CaCO_3_ content (550 g kg^−1^, which was the highest among the soils). Applying both nutrients (P and Zn) in combination to INM improved maize performance to a certain extent; thus, plant height was similar to that obtained in LCV and FER but only two maize plants grown on INM produced any grains—in both cases, the soil was supplied jointly with P and Zn.

As shown by our results, maize plants grown on Mediterranean soils containing limited amounts of available Zn must be supplied with P and Zn in combination for adequate development. These nutrients are known to interact negatively not only in soil but also within plants, and to alter plant morphology and physiology as a result^24^. Also, application of P or Zn alone impaired maize growth, probably because it disrupted the balance between these essential nutrients in the soil—or within plants following uptake. Treatment *P* reduced plant height but increased stem diameter and number of leaves, which is consistent with reported changes in wheat grown on low-Zn soils by effect of P fertilization^25^ but contradicts the results of some studies where P fertilization boosted plant growth^26,27^. On the other hand, supplying the soil with Zn only had a slight effect on plant growth relative to the control plants—which received no P or Zn—irrespective of the high initial available P content of the three soils (> 15 mg kg^−1^). Conversely, all plant variables assessed were favourably affected by the joint application of P and Zn, which resulted in a significant increase in plant biomass (30–60% in LCV, 48–62% in FER and 70–90% in INM) relative to application of either or neither nutrient. In line with this result, treatment *PZn* boosted yield (31% in LCV and 121% in FER), whereas treatments *P* and *Zn* diminished maize yield (by 80% in LCV and 8–63% in FER). Under growth-limiting conditions such as those provided by the studied soils, joint application of both nutrients (P and Zn) seems to provide an effective means for minimizing negative interactions between them as well as for increasing their availability to maize plants.

The basic pH of the three soils (near 8.0), and their content in calcite and in OM, restrict P and Zn availability and uptake by plants^4^. While application of P or Zn alone increased absorption of these nutrients in some plant parts^3^, the total amount of P and Zn in grain at harvest was reduced in relation to the control plants except when P and Zn were applied jointly to the soil. Therefore, P had an antagonistic effect on plant Zn gain over the control plants—so much so that gains were negative on LCV and FER. Excessive soil P content and application of P may reduce soil Zn phytoavailability^27^ and cause Zn immobilization in roots; also, it can restrict translocation of Zn by effect of the excess of P in plants because of the formation of Zn phytate and phosphate in the root apoplast^28^. Therefore, too much P in the soil can adversely affect Zn uptake and translocation^29^. Moreover, P fertilization is known to reduce plant symbiosis with arbuscular mycorrhizae, which provide a source of additional Zn by increasing the soil volume that can be explored by plants^25^.

Applying Zn but no P to the soils had an adverse effect on P gain over the control plants. Unlike Manzeke et al.^30^, who used a combination of chemical and organic fertilizers, we found no effect on grain P concentrations. In fact, the joint application of P and Zn was the only treatment increasing grain P and Zn uptake—in soils LCV and FER—, probably because *PZn* was the sole treatment affording adequate soil P and Zn availability despite the adverse impact of their interaction in soil and plant tissues.

Subsequently, the distribution of P and Zn in the different plant parts was similar in the control plants and in those treated with both nutrients—which exhibit increased P and Zn accumulation in grains. This was not the case with the plants supplied with a single nutrient (P or Zn), where P and Zn accumulated largely in leaf, stem and corncob. This result provides further evidence that fertilization with P or Zn alone causes an imbalance in these nutrients in plants. This was especially so in soils LCV and FER—in fact, only 2 of the 16 plants grown on INM produced any grains. Santos et al.^31^ previously found an appropriate plant Zn status to be needed on application of P to avoid nutritional problems—which seemingly occurred unless P and Zn were applied together.

Or results suggest that the P:Zn ratio in leaf, stem or corncob leaf, in combination with P and Zn uptake, may provide a powerful predictor for adequate translocation of these two nutrients to grains. Thus, a low ratio, in combination with an adequate P and Zn uptake, can be expected to maximize translocation of both elements, and hence to increase grain P and Zn use efficiency. These results are consistent with those of Kutman et al.^14^, who found accumulation of Zn in stem to be essential for subsequent remobilization to grains. However, we found accumulation of Zn in the stem of *Zn* plants not to be the only factor involved. We also found that Zn mobilization to grains can be impaired by a limited plant uptake of P under these conditions as previously seen by Zhang et al^3^.

The decreased grain P concentration found in plants grown on FER fertilized with P and Zn in combination (58% relative to the control plants) can be partially ascribed to a dilution effect. Thus, the previous plants produced 120–500% more grain than did *C* plants and those fertilized with P or Zn alone. This was not the case with the plants grown on LCV, probably because the properties of this soil (particularly its low carbonate content relative to the other soils) were not as limiting.

The grain Zn content of the control plants was typical of non-biofortified maize^30^ and, unexpectedly, higher in FER (32.1 mg kg^−1^) than it was in LCV (23.1 mg kg^−1^). Although it increased yield, supplying P and Zn in combination to LCV and FER had no dilution effect on grain Zn concentrations. In fact, such concentrations were around 36 mg kg^−1^ in LCV and FER, and 48 mg kg^−1^ in INM, both of which are similar to the target value for biofortified maize (> 38 mg kg^−1^^32^). Also, the low P:Zn ratios in grain from plants grown on LCV or FER fertilized jointly with P and Zn relative to the control plants suggest an increased Zn bioavailability^3^—and a potential benefit for human and animal health as this reduces the phytate content of grain.

Previous studies found excessive P fertilization to decrease grain Zn concentrations in cereals by 20–60%^33^. However, the relatively high grain P and Zn concentrations of the plants grown on LCV and FER supplied with a single nutrient (P or Zn) were due to limited grain production in relation to the other plants (viz., those that received both nutrients or neither). This result provides further evidence for the changes induced by fertilizing maize with P or Zn alone under these conditions. As shown here, applying P and Zn in combination had a favourable effect on nutrient uptake, yield and grain quality (Zn concentration, at least in LCV and INM).

The proteomic approach used here (shotgun, LC–MS/MS) enabled the identification and quantification of a large set of proteins in mature maize grains altered by fertilization with P and Zn, whether individually or in combination. Although we only focused on the proteins associated to grain nutritional quality, we examined all identified and quantified. Also, although the total amount of protein was not altered by any fertilizer treatment, the amounts of individual proteins did differ depending on the nutrient supplied^15^.

The increased amounts of globulins found in *Zn* (globulin-2, globulin-1 S allele, vicilin-like seed storage and legumin1) and *PZn* plants (globulin-1 S allele and vicilin-like seed storage) is known to increase grain quality by increasing the contents in lysine, methionine and cysteine^34^. Zn fertilization also altered the amounts of other less abundant grain storage proteins such as zeins and glutelins. In previous studies, γ-zeins were found to increase by effect of a decrease in α-zeins, and also to modify the endosperm structure of Quality Protein Maize (QPM) lines as a result^35^. Although the major 19-kDa and 22-kDa α-zeins exhibited no significant differences between fertilizer treatments, they were less abundant in *Zn* plants than in *P* plants or even *PZn* plants. Previous studies showed removing 19-kDa and 22-kDa α-zeins to increase lysine and tryptophan contents^36^.

Application of P and Zn altered not only grain storage proteins, but also proteins involved in other biological processes potentially related to nutritional quality. Thus, fertilization with Zn or, to a lesser extent, P and Zn, significantly increased the levels of some proteins such as glyceraldehyde-3-phosphate dehydrogenase and enolase1, which are associated to carbohydrate metabolism. Late embryogenesis abundant (LEA) and heat shock proteins (HSPs), together with grain storage proteins, provide tolerance to grain desiccation after long-term storage and increase survival rates by helping retain germination ability under dry conditions^37^. Oil body-associated protein 1A, which plays a major role in the biogenesis of oil bodies, has been found to decrease the germination ratio and seed oil content in *Arabidopsis thaliana*^38^. The hydroxyproline-rich glycoprotein family was the fifth most abundant protein identified here. Because this protein family is rich in hydroxyproline, threonine, proline, lysine and glycine, an increase in its content by effect of treatment *Zn* or even *PZn* could increase the proportion of lysine in maize grain^39^. Also, this protein is found in the wall of maize pericarp, which is a protective and supportive tissue^39^. The levels of NAD(P)-binding Rossmann-fold protein superfamily, which is associated to anthocyanin biosynthesis in the pericarp and aleurone layer in maize^40^, were also increased by fertilization. Therefore, application of Zn and, to a lesser extent, P and Zn, increased the quality of maize grains in term of seed storage, lys-rich and grain wall proteins. The fact that grain yield was not increased in *Zn* plants is consistent with the results of previous studies on maize improved lines where maize quality was negatively correlated with grain yield^41^. Further research is therefore needed to identify the best trade-off between grain yield and protein quality in maize grown on alkaline Mediterranean soils fertilized with P and/or Zn.

**Figure 3:**
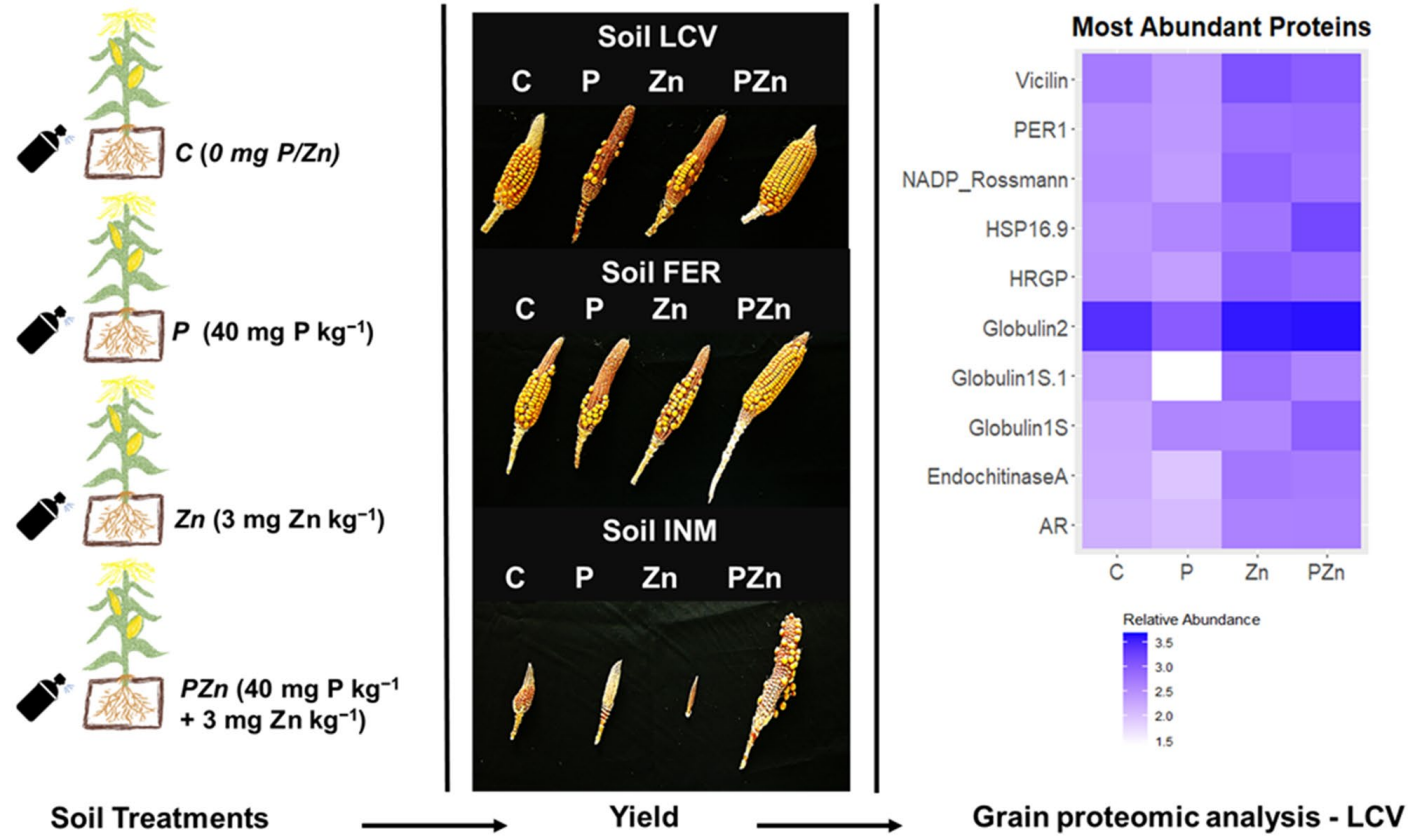
Schedule summarizing the effects of P and/or Zn application to the soil on yields of maize plants grown on Soil FER, LCV and INM and on relative abundance of the most abundant proteins in grain at harvest (Soil LCV; heatmap adapting code from https://jcoliver.github.io/learn-r/006-heatmaps.html).

Our results expand existing knowledge about the P–Zn interaction in maize grown on Mediterranean soils and underline the importance of using P in combination with Zn to fulfil the requirements of so highly demanding C4-type plants such as maize. Supplying only one of the nutrients hinders P and Zn uptake, and, especially, P and Zn translocation from leaf and stem to grain. As shown here, application of P or Zn alone restricted maize growth (plant height, stem diameter or plant biomass) and reduced yield (by 8 to 85% relative to control plants) in soils with medium to high P contents and low Zn contents (Fig. 3). The joint application of both nutrients increased plant biomass (30% in LCV, 50% in FER and 257% in INM), yield (31% in LCV and 121% in FER) and Zn grain concentration (more than 55% in LCV) relative to the control plants receiving no P or Zn. Also, the grains from plants fertilized with both P and Zn had lower P:Zn ratios—and thus contained more available Zn for human or animal consumption—than the plants that were not fertilized with any of these nutrients. The proteomic profile for mature grains suggested that application of Zn or, to a lesser extent, both nutrients, increased maize grain quality by increasing the abundance of seed storage (globulins), lys-rich (glyceraldehyde-3-phosphate dehydrogenase and enolase1) and cell wall proteins (hydroxyproline-rich glycoprotein family and NAD(P)-binding Rossmann-fold protein superfamily; Fig. 3).

Applying P and Zn in combination to Mediterranean soils with limited Zn availability is therefore essential to increase maize yields or even grain quality through an increased Zn availability and grain protein quality. These findings should be considered in developing sustainable strategies for agriculture in Mediterranean areas and, indeed, in any others where soil P and Zn availability are restricted by a basic pH and an unfavourable mineralogy (too high a CaCO_3_ content). Further research is needed, however, to identify the optimum P:Zn ratio to be used when fertilizing maize under these conditions, as well as the legacy effects of these practices on P and Zn phytoavailability, and grain yield and quality.

## Supplementary Information

Supplementary Information 1.

Supplementary Information 2.

Supplementary Information 3.

Supplementary Information 4.

Supplementary Information 5.

Supplementary Information 6.

## Data Availability

The mass spectrometry proteomics data have been deposited to the ProteomeXchange Consortium via the PRIDE partner repository with the dataset identifier PXD021503.
