# Supplementary material for: Combining P and Zn fertilization to enhance yield and grain quality in maize grown on Mediterranean soils
Source: Sci Rep. 2021 Apr 1;11:7427. doi: 10.1038/s41598-021-86766-2 (PMC8016957; doi:10.1038/s41598-021-86766-2)
Supplement: Supplementary file 1 — Supplementary Information 1. [file 41598_2021_86766_MOESM1_ESM.pdf]

Combining P and Zn fertilization to enhance yield and grain quality in maize grown on Mediterranean soils

Scientific Reports

Antonio Rafael Sánchez-Rodríguez[1], María-Dolores Rey[2], Hasna Nechate-Drif[1], María Ángeles Castillejo[2], Jesús V. Jorrín-Novo[2], José Torrent[1], María Carmen del Campillo[1], Daniel Sacristán[1]

[1] Department of Agronomy, University of Córdoba, Campus de Rabanales, Building C4, 14071 Córdoba, Spain

[2] Department of Agroforestry and Plant Biochemistry, Proteomics and Systems Biology, Biochemistry and Molecular Biology, University of Córdoba, Campus de Rabanales, Building C6, 14071 Córdoba, Spain

*Corresponding authors: email addresses: antonio.sanchez@uco.es (AR Sánchez-Rodríguez). Phone: +34 957 21 21 83 and b52resam@uco.es (M-D Rey). Phone: +34 957 21 85 74

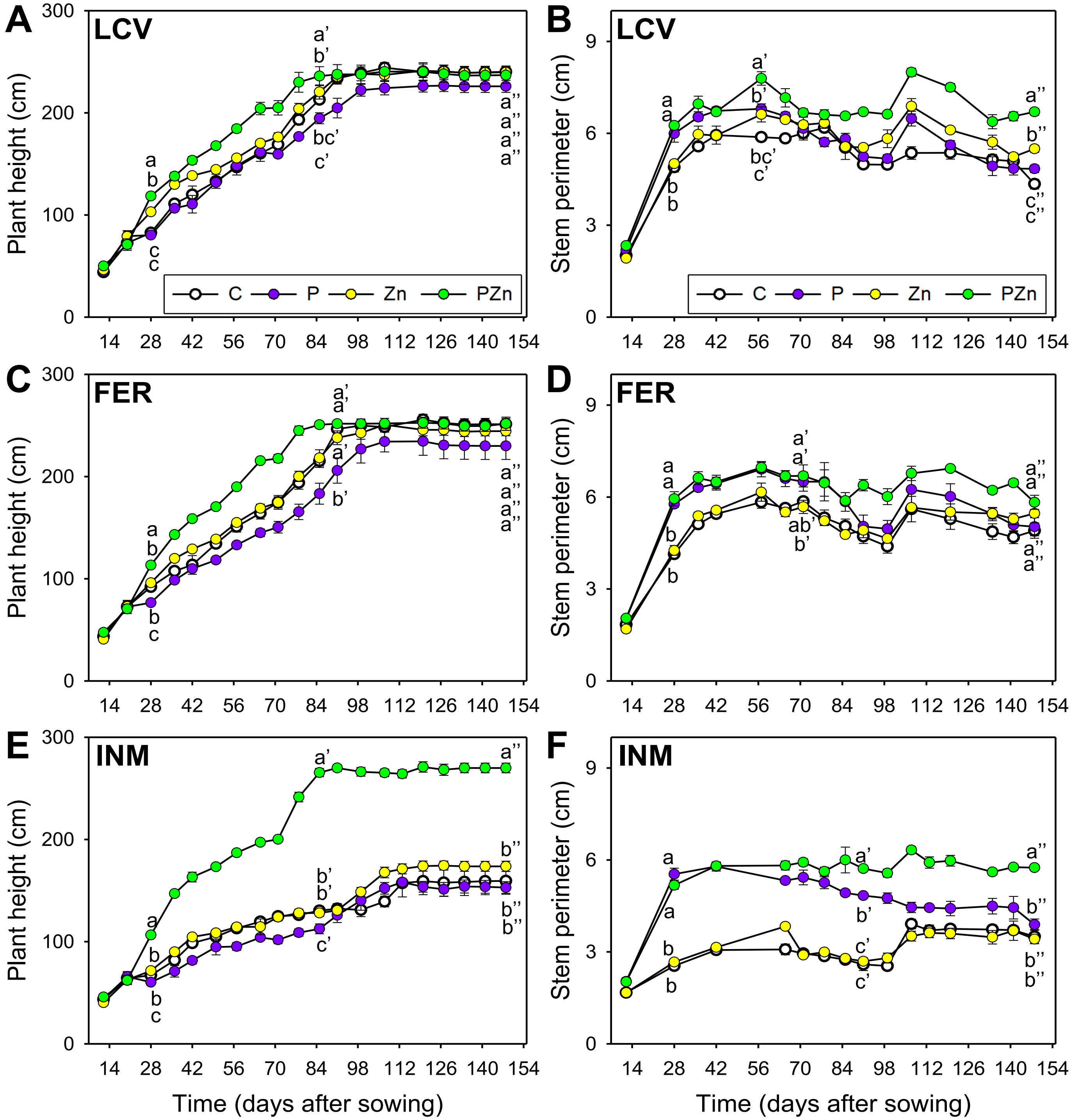

Fig. S1 Time course of plant height and stem perimeter (mean ± standard error, *n* = 4) as a function of soil (**A** and **B**, LCV; **C** and **D**, FER; **E** and **F**, INM) and treatment (*C:* no P or Zn was added; *P:* fertilization with 40 mg P kg^{-1} but no Zn; *Zn:* fertilization with 3 mg Zn kg^{-1} but no P; *PZn:* fertilization with 40 mg P kg^{-1} and 3 mg Zn kg^{-1}). Different lowercase letters indicate significant differences among treatments at different times as per the post-hoc LSD test. Although all data were analyzed, only the results of three analyses are shown due to the high frequency of measurements (viz., those where different trends were observed during the experiment).

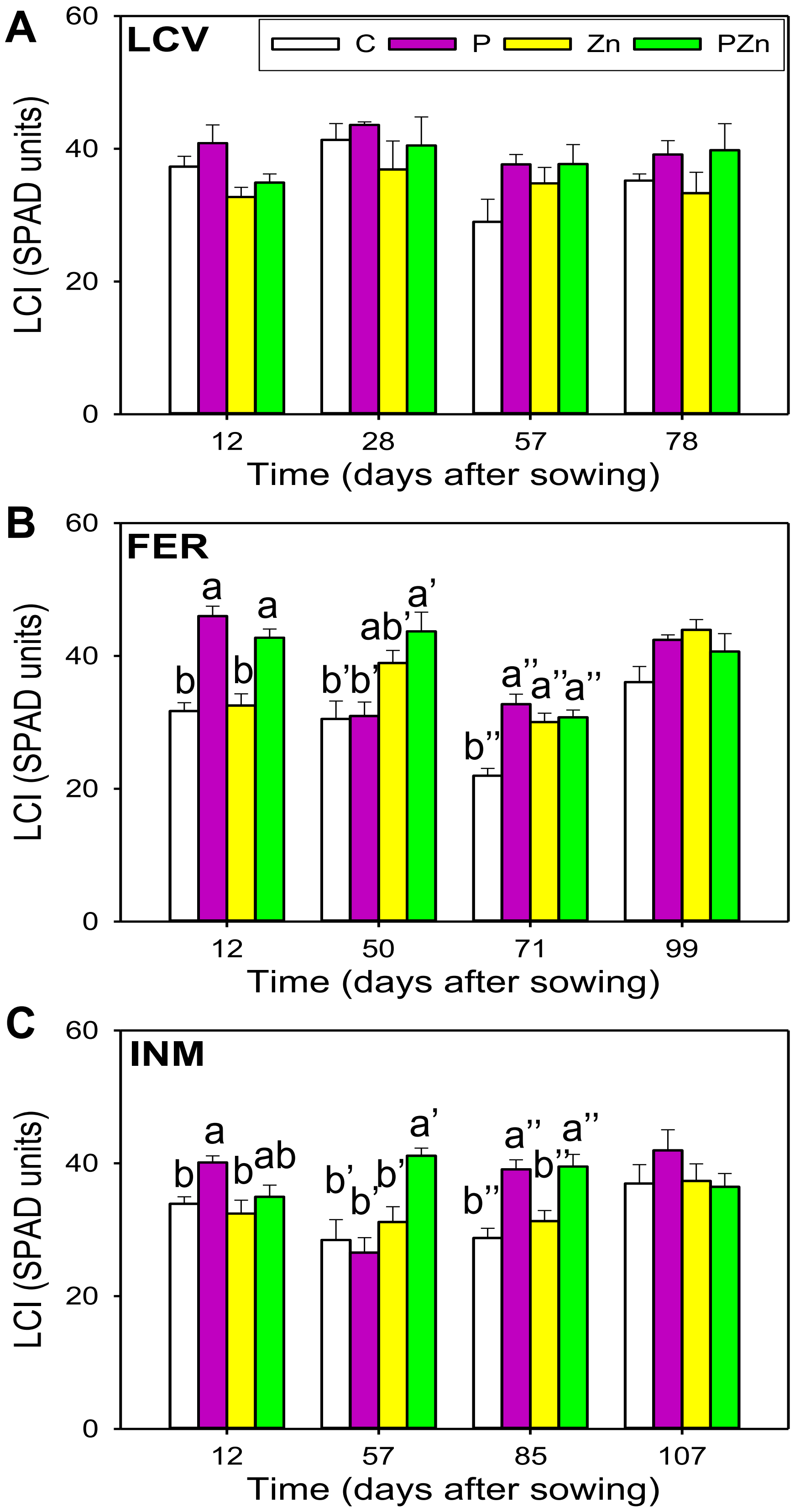

Fig. S2 Time course of leaf chlorophyll index (LCI, SPAD units; mean ± standard error, *n* = 4) as a function of soil (**A**, LCV; **B**, FER; **C**, INMC) and treatment. na: not available. Different lowercase letters indicate significant differences between treatments at different times as per the post-hoc LSD test. No letter for a specific day means there were no significant differences.

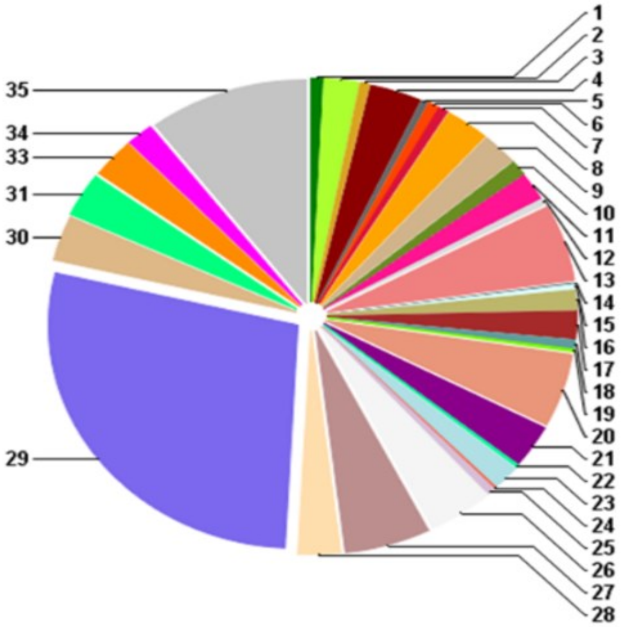

1- PS	0.74%	2- major CHO metabolism	2.23%
3- minor CHO metabolism	0.58%	4- glycolysis	3.31%
5- fermentation	0.41%	6- gluconeogenesis /glyoxylate cycle	0.74%
7- OPP	0.58%	8- TCA / org transformation	2.81%
9- mitochondrial electron transport / ATP synthesis	2.31%	10- cell wall	1.07%
11- lipid metabolism	1.98%	12- N-metabolism	0.41%
13- amino acid metabolism	5.54%	14- S-assimilation	0.08%
15- metal handing	0.41%	16- secondary metabolism	1.40%
17- hormone metabolism	1.98%	18- Co-factor and vitamine metabolism	0.66%
19- tetrapyrrole synthesis	0.17%	20- stress	5.29%
21- redox	3.06%	22- polyamine metabolism	0.17%
23- nucleotide metabolism	1.82%	24-Biodegradation of Zenobiotics	0.17%
25- C1-metabolism	0.50%	26- misc	4.13%
27- RNA	5.54%	28- DNA	2.73%
29- protein	27.93%	30- signalling	3.22%
31- cell	3.14%	33- development	2.81%
34- transport	1.74%	35 not assigned	10.33%

Fig. S3 Molecular function analysis of the differentially abundant proteins (DAPs) identified here.
