# Supplementary material for: Combining P and Zn fertilization to enhance yield and grain quality in maize grown on Mediterranean soils
Source: Sci Rep. 2021 Apr 1;11:7427. doi: 10.1038/s41598-021-86766-2 (PMC8016957; doi:10.1038/s41598-021-86766-2)
Supplement: Supplementary file 2 — Supplementary Information 2. [file 41598_2021_86766_MOESM2_ESM.pdf]

Combining P and Zn fertilization to enhance yield and grain quality in maize grown on Mediterranean soils

Scientific Reports

Antonio Rafael Sánchez-Rodríguez[1], María-Dolores Rey[2], Hasna Nechate-Drif[1], María Ángeles Castillejo[2], Jesús V. Jorrín-Novo[2], José Torrent[1], María Carmen del Campillo[1], Daniel Sacristán[1]

[1] Department of Agronomy, University of Córdoba, Campus de Rabanales, Building C4, Crta. Madrid, Km 396, 14071 Córdoba, Spain

[2] Department of Agroforestry and Plant Biochemistry, Proteomics and Systems Biology, Biochemistry and Molecular Biology, University of Córdoba, Campus de Rabanales, Building C6, Crta. Madrid, Km 396, 14071 Córdoba, Spain

*Corresponding authors: email addresses: antonio.sanchez@uco.es (AR Sánchez-Rodríguez). Phone: +34 957 21 21 83 and b52resam@uco.es (M-D Rey). Phone: +34 957 21 85 74

Table S1 Soil properties (mean of two duplicates).

Soil	Sand	Silt	Clay	OM	CEC	$EC_{1:5}$	$pH_{1:2.5}$	CO_3^{-2}	Fe_{ox}	P_{Olsen}	Zn_{DTPA}
	g kg^{-1}	g kg^{-1}	g kg^{-1}	g kg^{-1}	$cmol_{c+}$ kg^{-1}	dS m^{-1}		g kg^{-1}	mg kg^{-1}	mg kg^{-1}	mg kg^{-1}
LCV	465	109	426	5.4	24.6	0.295	7.79	43	0.82	15.5	0.51
FER	503	198	298	4.7	32.0	0.175	7.89	339	0.49	17.9	0.48
INM	634	195	173	2.9	13.4	0.135	7.96	552	0.20	19.7	0.46

OM: organic matter; CEC: cation exchange capacity; EC: electrical conductivity of the 1:5 soil:water extract; CO_3^{-2}: carbonates; Fe_{ox}: poorly crystalline Fe oxides extracted with ammonium oxalate; P_{Olsen}: soil available P extracted with $NaHCO_3$; Zn_{DTPA}: soil available Zn extracted with DTPA (diethylenetriamine penta-acetic acid).

Material and methods

Soil texture was analysed by the pipette method after dispersion with Na hexametaphosphate, organic carbon (OC) by rapid dichromate oxidation, total $CaCO_3$ equivalent by weight loss after 6 M HCl addition (van Wesemael, 1951) and cation exchange capacity (CEC) after cation extraction with 1M ammonium acetate buffered at pH 7.0. Soil pH was measured by potentiometry in a 1:2.5 soil:water suspension and soil electrical conductivity (EC) in a 1:5 soil:water suspension with a conductivity meter. Available P (P_{Olsen}) was determined by the molybdenum blue method (Murphy & Riley, 1962) after extraction with 0.5 M $NaHCO_3$ buffered at pH 8.5 (Olsen, Cole, Watanabe, & Dean, 1954). Available zinc (Zn_{DTPA}) was measured by using atomic absorption spectroscopy after extraction with diethylenetriaminepentaacetic acid-extractable (Lindsay & Norvell, 1978). Finally, poorly crystalline iron oxides (Fe_{ox}) were determined after extraction with ammonium oxalate at pH 3.0 following Schwertmann (1964), except for the soil:solution ratio (1:200) to prevent alterations in the pH due to the dissolution of soil carbonates in the process (Benítez, Pedrajas, del Campillo, & Torrent, 2002).

Results

The highest percent in sand (63.4%) and the lowest in clay (17.3%) were measured in INM (sandy loam texture), while the highest percent in clay was for LCV (42.6%; sandy clay texture) followed by FER (29.8%, sandy clay loam texture; Table 1). Organic carbon was low in all of them (<6 g kg^{-1}), especially in INM (<3 mg kg^{-1}), while CEC ranged

between 13.4 (INM) and 32.0 (FER) $cmol_{c+}$ kg^{-1}. Soil pH was near 8 in the three soils due to the presence of carbonates (43 g kg^{-1} in LCV, 339 g kg^{-1} in FER and 552 g kg^{-1} in INM; Table 1). The content in Fe_{ox} was very low in INM (0.20 mg kg^{-1}) and low in FER (0.49 mg kg^{-1}), but relatively high in LCV (0.82 mg kg^{-1}; Table 1). Available P (P_{Olsen}) was medium-high in all soils (15.5–19.7 mg kg^{-1}), while soil available Zn (Zn_{DTPA}) ranged between 0.46 (INM) and 0.51 mg kg^{-1} (LCV), near the critical value for deficiency in cereals (0.50 mg kg^{-1}; Lindsay & Norvell 1978).

References

Benítez, M. L., Pedrajas, V. M., del Campillo, M. C., & Torrent, J. (2002). Iron chlorosis in olive in relation to soil properties. *Nutrient Cycling in Agroecosystems*. https://doi.org/10.1023/A:1015116732580

Lindsay, W. L., & Norvell, W. A. (1978). Development of a DTPA soil test for zinc, iron, manganese and copper. *Soil Science Society of America Journal*, *42*, 421–428.

Murphy, J., & Riley, J. P. (1962). A modified single solution method for the determination of phosphate in natural waters. *Analytica Chimia Acta.*, *27*, 31–36.

Olsen, S. R., Cole, C. V., Watanabe, F. S., & Dean, L. A. (1954). Estimation of available phosphorus in soils by extraction with sodium bicarbonate. *USDA Circular*, 18. https://doi.org/10.2307/302397

Schwertmann, U. (1964). Differenzierung der Eisenoxide des Bodens durch Extraktion mit Ammoniumoxalat-Lösung. *Zeitschrift Für Pflanzenernährung, Düngung, Bodenkunde*. https://doi.org/10.1002/jpln.3591050303

van Wesemael, J. C. (1951). Debepaling van het calciumcarbonaat–gehalte van gronden. *Chemisch Weekblad*, 35–36.
