# Supplementary material for: Combining P and Zn fertilization to enhance yield and grain quality in maize grown on Mediterranean soils
Source: Sci Rep. 2021 Apr 1;11:7427. doi: 10.1038/s41598-021-86766-2 (PMC8016957; doi:10.1038/s41598-021-86766-2)
Supplement: Supplementary file 3 — Supplementary Information 3. [file 41598_2021_86766_MOESM3_ESM.pdf]

Combining P and Zn fertilization to enhance yield and grain quality in maize grown on Mediterranean soils

Scientific Reports

Antonio Rafael Sánchez-Rodríguez[1], María-Dolores Rey[2], Hasna Nechate-Drif[1], María Ángeles Castillejo[2], Jesús V. Jorrín-Novo[2], José Torrent[1], María Carmen del Campillo[1], Daniel Sacristán[1]

[1] Department of Agronomy, University of Córdoba, Campus de Rabanales, Building C4, 14071 Córdoba, Spain

[2] Department of Agroforestry and Plant Biochemistry, Proteomics and Systems Biology, Biochemistry and Molecular Biology, University of Córdoba, Campus de Rabanales, Building C6, 14071 Córdoba, Spain

*Corresponding authors: email addresses: antonio.sanchez@uco.es (AR Sánchez-Rodríguez). Phone: +34 957 21 21 83 and b52resam@uco.es (M-D Rey). Phone: +34 957 21 85 74

Table S2 Repeated measures ANOVA for plant height, stem perimeter, number of leaves, leaf thickness and leaf chlorophyll index (LCI, SPAD units; mean, *n* = 16 for each treatment and *n* = 48 for each sampling time) for maize plants grown on soils LCV, FER and INM. Different letters indicate significant differences ($p < 0.05$) as per the post-hoc LSD test. (*C* no P or Zn was added; *P* fertilization with 40 mg P kg^{-1} but no Zn; *Zn* fertilization with 3 mg Zn kg^{-1} but no P; *PZn* fertilization with 40 mg P kg^{-1} and 3 mg Zn kg^{-1}). –: not available.

		Plant height (cm)	Stem perimeter (mm)	Leaf number	Leaf thickness (mm)	LCI (SPAD units)
			**LCV soil**			
Treatment	C	177	52	14	0.166	35.2
	P	168	57	15	0.174	38.2
	Zn	183	57	14	0.170	34.8
	PZn	194	67	14	0.180	38.9
	p-value	0.0250	0.0001	0.0127	0.0075	0.1216
Time (days after sowing)	12	46	21	4	0.169	36.4 ABCD
	20	74	-	7	-	41.9 A
	28	99	56	9	0.187	40.6 ABC
	36	121	63	11	0.186	34.9 BCDE
	42	134	64	13	0.170	32.1 DE
	50	144	67	14	0.187	36.2 ABCD
	57	159	68	15	0.175	34.8 CDE
	65	173	65	15	0.172	34.9 BCDE
	71	179	63	16	0.165	28.4 E
	78	201	62	16	0.174	36.9 ABCD
	85	216	59	17	0.173	41.5 AB
	91	228	56	17	0.148	38.5 ABCD
	99	234	56	17	0.167	38.0 ABCD
	107	235	57	17	0.172	39.8 ABC
	120	235	56	17	-	-
	127	235	-	17	-	-
	134	236	54	-	-	-
	141	237	54	-	-	-
	148	238	53	-	-	-
	p-value	0.0000	0.0000	0.0000	0.0000	0.0000
Interaction	*p*-value	0.0000	0.0000	0.0000	0.0000	0.1528
			**FER soil**			
Treatment	C	183	50	14	0.167	35.2
	P	164	57	14	0.170	37.9
	Zn	183	51	13	0.171	37.6
	PZn	203	62	13	0.183	38.3
	p-value	0.0033	0.0013	0.1483	0.0180	0.0648
Time (days after sowing)	12	44	19	4	0.167	38.2
	20	72	-	6	-	39.8
	28	96	50	8	0.184	42.0
	36	117	59	11	0.187	33.4
	42	130	60	12	0.178	30.9
	50	140	63	13	0.184	36.0
	57	157	65	14	0.168	34.4
	65	173	61	15	0.170	35.3
	71	182	62	15	0.167	28.9
	78	201	59	16	0.170	38.7
	85	217	54	16	0.169	41.0
	91	235	53	17	0.159	40.3

	99	242	50	17	0.175	40.8
	107	245	61	17	0.168	41.7
	120	246	59	17	-	-
	127	247	-	17	-	-
	134	247	55	-	-	-
	141	247	54	-	-	-
	148	247	54	-	-	-
	p-value	0.0000	0.0000	0.0000	0.0000	0.0000
Interaction	*p*-value	0.0000	0.0087	0.0000	0.0000	0.0001
			**INM soil**			
Treatment	Control	120	31	10	0.143	30.0
	Phosphorus	111	48	13	0.188	35.6
	Zinc	127	31	10	0.140	30.7
	Phosphorus+zinc	207	56	13	0.179	38.0
	p-value	0.0000	0.0000	0.0000	0.0000	0.0006
Time (days after sowing)	12	43	18	4	0.166	35.4
	28	78	40	7	0.171	37.8
	36	99	47	9	0.179	35.3
	42	112	44	10	0.177	31.0
	50	119	45	11	0.172	33.3
	57	128	46	12	0.162	31.8
	65	134	43	12	0.156	31.7
	71	139	43	13	0.149	26.6
	78	151	42	13	0.157	34.1
	85	159	41	14	0.150	34.7
	91	164	40	15	0.153	33.5
	99	173	45	15	0.158	32.7
	107	182	45	15	0.162	38.7
	120	188	44	15	-	-
	127	189	43	15	-	-
	134	189	43	-	-	-
	141	190	44	-	-	-
	148	190	41	-	-	-
	p-value	0.0000	0.0000	0.0000	0.0000	0.0000
Interaction	*p*-value	0.0000	0.0000	0.0000	0.0000	0.0056
