# Supplementary material for: Combining P and Zn fertilization to enhance yield and grain quality in maize grown on Mediterranean soils
Source: Sci Rep. 2021 Apr 1;11:7427. doi: 10.1038/s41598-021-86766-2 (PMC8016957; doi:10.1038/s41598-021-86766-2)
Supplement: Supplementary file 5 — Supplementary Information 5. [file 41598_2021_86766_MOESM5_ESM.pdf]

Combining P and Zn fertilization to enhance yield and grain quality in maize grown on Mediterranean soils

Scientific Reports

Antonio Rafael Sánchez-Rodríguez[1], María-Dolores Rey[2], Hasna Nechate-Drif[1], María Ángeles Castillejo[2], Jesús V. Jorrín-Novo[2], José Torrent[1], María Carmen del Campillo[1], Daniel Sacristán[1]

[1] Department of Agronomy, University of Córdoba, Campus de Rabanales, Building C4, 14071 Córdoba, Spain

[2] Department of Agroforestry and Plant Biochemistry, Proteomics and Systems Biology, Biochemistry and Molecular Biology, University of Córdoba, Campus de Rabanales, Building C6, 14071 Córdoba, Spain

*Corresponding authors: email addresses: antonio.sanchez@uco.es (AR Sánchez-Rodríguez). Phone: +34 957 21 21 83 and b52resam@uco.es (M-D Rey). Phone: +34 957 21 85 74

Table S4 List of all the proteins grouped in the most abundant proteins, seed storage proteins and trp-rich and lys-rich proteins. Proteins identified from statistically significant differences between samples in an ANOVA (*p* ≤ 0.05) and fold ≥ 2 or ≤ 0.5. The ID and description, number of peptides (≥ 2), score (≥ 2) and coverage (≥ 15%), relative abundance (%), *p* value (≤ 0.05) and ratio (fold ≥ 2 or ≤ 0.5) of each protein is shown.

Protein ID	Description	No. of peptides	Score Sequest	Coverage	%	*p-value*	Ratio		
							P/C	ZN/C	PZn/C
**Most abudant proteins**									
Q7M1Z8	Globulin-2	34	9504.25	74.22	15.39	*0.0243*	0.30	1.94	0.89
K7W272	Vicilin-like seed storage	31	5143.54	59.05	3.5	*0.0351*	0.50	2.68	1.27
B6SIX0	16.9 kDa class I heat shock protein 1	10	1559.87	68.18	3.27	*0.3273*	0.45	1.99	2.09
A0A1D6K1B9	Hydroxyproline-rich glycoprotein family protein	25	3469.82	73.83	2.48	*0.0327*	0.50	2.88	1.26
A0A1D6F0W7	NAD(P)-binding Rossmann-fold superfamily protein	25	2708.60	74.86	2.47	*0.0304*	0.49	2.59	1.13
C0PGM3	Globulin-1 S allele	48	9146.75	71.15	2.26	*0.1063*	1.22	2.89	4.41
A2SZW8	1-Cys peroxiredoxin PER1	17	1719.43	74.24	2.11	*0.1216*	0.49	2.20	1.34
P15590	Globulin-1 S allele	44	8140.84	64.75	1.8	*0.0022*	0.26	3.31	1.34
B4FTS6	Endochitinase A	17	1598.83	80.00	1.46	*0.0574*	0.46	2.89	1.45
E9JVD4	Aldose reductase	27	1514.19	75.24	1.27	*0.1201*	0.52	2.60	1.42
Q43264	Alcohol dehydrogenase 1	21	2693.07	82.85	1.18	*0.13*	0.45	2.81	1.76
A0A1D6LER3	Glyceraldehyde-3-phosphate dehydrogenase	23	2381.58	79.94	1.02	*0.0363*	0.45	2.98	1.27
A0A1D6IYD6	Glyoxylase1	15	1188.43	59.21	0.91	*0.1154*	0.61	3.21	1.83
B4G0K4	Phosphoglycerate kinase	33	2199.91	86.82	0.88	*0.0596*	0.45	2.62	1.31
B4FFZ9	Oil body-associated protein 1A	12	882.02	63.87	0.83	*0.0395*	0.50	2.36	1.08
B4FUH2	Aspartate aminotransferase	24	2103.98	61.22	0.79	*0.1009*	0.51	2.54	1.43
K7V794	Enolase 1	31	2874.43	80.49	0.75	*0.0386*	0.51	3.10	1.46
K7VJF3	Heat shock 70 kDa protein 5	46	2773.17	72.24	0.69	*0.0023*	0.27	3.33	0.46
A0A1D6HCF4	Glyceraldehyde-3-phosphate dehydrogenase4	18	1494.59	47.84	0.65	*0.0566*	0.53	3.64	1.71
B6UH67	Late embryogenesis abundant protein D-34	13	1494.92	57.52	0.59	*0.0099*	0.43	3.11	0.95
B6SK87	rRNA N-glycosidase	18	1375.68	74.42	0.57	*0.0169*	0.33	3.10	1.18
A0A1D6N932	Osmotin-like protein OSM34	13	627.81	74.55	0.53	*0.12*	0.87	5.10	2.03

B4FE26	11-beta-hydroxysteroid dehydrogenase 1A	15	1297.12	57.59	0.53	*0.062*	0.36	3.12	1.60
A0A1D6NT56	Sucrose synthase	45	2475.12	54.50	0.51	*0.0352*	0.28	2.59	0.85
**Seed storage proteins**									
Q7M1Z8	Globulin-2	34	9504.25	74.22	15.39	*0,0243*	0.30	1.94	0.89
C0PGM3	Globulin-1 S allele	1.19	3.72	1.44	2.26	*0,1063*	1.22	2.89	4.41
P15590	Globulin-1 S allele	44	8140.84	64.75	1.80	*0,0022*	0.26	3.31	1.34
A0A1R3QMY2	50kD gamma zein	7	552.98	36.04	0.43	*0,0292*	0.39	2.32	0.48
P06673	Zein-beta	0.19	0.41	0.17	0.23	*0,3574*	0.83	2.06	2.10
Q946V7	19kD alpha zein D2	0.12	0.33	0.07	0.20	*0,4930*	1.09	1.49	5.15
Q946W0	15kD beta zein	0.04	0.2	0.03	0.09	*0,1430*	1.84	1.52	6.64
Q946V3	Alpha globulin	0.07	0.04	0.04	0.05	*0,1236*	0.22	1.52	0.95
P04706	Glutelins	2	149.51	12.11	0.03	*0,0022*	1.39	0.36	0.93
P04701	Zein	2	75.44	26.22	0.03	-	1.44	0.00	3.11
A0A1D6G9W0	2S albumin[a]	0.02	0.01	0.01	0.02	*0,5409*	0.18	1.63	3.22
P04698	22 kDa alpha-zein 14	0.02	0.05	0.01	0.02	*0,2053*	0.87	1.11	1.24
A0A1D6KYZ7	Globulin-1 S allele	24	2581.57	81.00	0.02	-	0.00	3.31	0.00
**Trp and Lys rich proteins**									
K7W272	Vicilin-like seed storage protein	31	5143.54	59.05	3.50	*0,0373*	0.55	2.76	2.74
A0A1D6LER3	Glyceraldehyde-3-phosphate dehydrogenase (EC 1.2.1.-)	23	2381.58	79.94	1.02	*0,0002*	0.33	3.48	0.00
A0A1D6NT56	Sucrose synthase (EC 2.4.1.13)	45	2475.12	54.50	0.51	*0,0243*	0.36	1.23	0.94
Q946V2	Legumin 1 (Legumin1)	23	1511.43	63.98	0.45	*0,0000*	0.24	2.95	0.37
A0A1D6K268	Vicilin-like seed storage protein	16	1089.95	38.52	0.36	-	-	-	-
Q5EUE1	Protein disulfide-isomerase (EC 5.3.4.1)	37	2027.87	71.40	0.34	*0,0334*	0.28	2.21	0.51
B4FAL9	Fructose-bisphosphate aldolase (EC 4.1.2.13)	35	2480.04	85.63	0.24	*0,0143*	0.31	3.73	0.90
A0A1D6KL30	Sorbitol dehydrogenase	18	961.14	68.03	0.20	*0,0007*	0.25	1.72	1.24
Q84TL6	Legumin-like protein (RmlC-like cupins superfamily protein)	15	927.24	74.10	0.19	*0,0635*	0.48	1.74	1.42
K7UUB7	Elongation factor 1-alpha	21	2688.99	57.05	0.18	-	0.29	2.78	0.68
A0A1D6FW13	Actin-7	19	1169.94	54.85	0.11	-	0.00	1.61	3.24

O50018	Elongation factor 1-alpha	20	1972.30	53.24	0.10	*0,0270*	0.29	0.94	0.79
Q5EUE0	Protein disulfide-isomerase (EC 5.3.4.1)	27	766.07	54.88	0.10	*0,0365*	0.58	1.40	1.89
B6UHJ4	Elongation factor 1-alpha	20	1642.73	53.24	0.09	-	0.00	1.33	2.57
B6SMQ5	Triose phosphate isomerase5	13	412.68	63.67	0.08	*0,0031*	1.09	1.49	5.15
A0A1D6K2D7	Sucrose synthase (EC 2.4.1.13)	35	758.50	45.11	0.07	*0,0241*	0.64	3.67	1.90
A0A1D6LZ74	Protein disulfide isomerase7	18	426.66	50.53	0.02	*0,0350*	0.72	2.69	1.96
B4FVB1	Actin-7	16	780.68	56.50	0.02	*0,0263*	0.26	1.45	1.42
Q5EUD5	Protein disulfide isomerase8	11	241.78	36.45	0.02	*0,0351*	0.47	3.81	1.01
B4FS35	Indole-3-glycerol phosphate synthase chloroplastic	9	194.28	29.24	0.02	0,0373	0.39	2.85	1.15
B6SLV6	16.9 kDa class I heat shock protein 3	4	122.89	38.26	0.01	-	-	-	-
B6TWN7	Elongation factor 1-alpha	17	826.50	39.15	0.01	*0,0107*	0.53	2.35	1.90
B6TQ08	Actin-1	18	894.63	66.05	0.01	*0,0363*	1.84	1.52	6.64
B4FAK8	Calnexin homolog2	11	227.60	26.69	0.01	*0,0412*	0.49	2.20	1.34
B4FQ44	L-tryptophan--pyruvate aminotransferase 1	5	92.76	16.01	0.01	0,0339	0.19	3.33	0.80
B4FNB4	Actin-related protein 4	7	76.96	19.41	0.01	*0,0695*	0.62	2.25	2.87
B4FRH8	Actin-7	18	905.21	66.05	0.01	*0,0033*	0.45	1.99	2.09
A0A1D6QSB0	Glyceraldehyde-3-phosphate dehydrogenase (EC 1.2.1.-)	14	519.20	53.78	0.01	*0,0012*	0.28	0.83	0.76
A0A1D6L7S0	Tryptophan synthase alpha chain chloroplastic	4	33.61	23.72	0.00	-	0.00	2.61	1.00
C0P6F8	Sucrose synthase (EC 2.4.1.13)	15	264.09	20.47	0.00	*0,0359*	0.64	3.50	1.39
A0A1D6ERC4	Protein disulfide-isomerase like 2-2	10	204.96	32.35	0.00	*0,0025*	0.42	2.15	0.78
K7TR93	Tryptophan synthase	6	36.29	17.95	0.00	-	0.62	0.43	0.00
Q5EUD1	Protein disulfide isomerase12	7	12.03	16.56	0.00	*0,0352*	0.87	1.11	1.24
A0A1D6M1H2	Elongation factor 1-alpha	8	555.30	51.09	0.00	-	0.36	3.12	1.60
